# Using motivational interviewing combined with digital shoe-fitting to improve adherence to wearing orthopedic shoes in people with diabetes at risk of foot ulceration: study protocol for a cluster-randomized controlled trial

**DOI:** 10.1186/s13063-021-05680-0

**Published:** 2021-10-28

**Authors:** M. Jongebloed-Westra, C. Bode, J. J. van Netten, P. M. ten Klooster, S. H. Exterkate, H. Koffijberg, J. E. W. C. van Gemert-Pijnen

**Affiliations:** 1 Department of Psychology, Health and Technology, Centre for eHealth Research and Wellbeing, TechMed Centre, University of Twente, PO Box 217, 7500 AE, Enschede, The Netherlands; 2 Department of Rehabilitation, Amsterdam UMC, location Academic Medical Centre, University of Amsterdam, Amsterdam Movement Sciences, Meibergdreef 9, 1105 AZ, Amsterdam, The Netherlands; 3 Diabetic Foot Unit, Department of Surgery, Hospital Group Twente, PO Box 7600, 7600 SZ, Almelo, The Netherlands; 4 Voetencentrum Wender, Sabina Klinkhamerweg 10, 7555 SK, Hengelo, The Netherlands; 5 Voetmax Orthopedie, Sabina Klinkhamerweg 10, 7555 SK, Hengelo, The Netherlands; 6 Department of Health Technology and Services Research, TechMed Centre, University of Twente, PO Box 217, 7500 AE, Enschede, The Netherlands

**Keywords:** Diabetes mellitus, Diabetic foot, Adherence, Behavior, Orthopedic shoes, Motivational interviewing, Cost-effectiveness, Patient satisfaction

## Abstract

**Background:**

Diabetic foot ulcers have a high impact on mobility and daily functioning and lead to high treatment costs, for example, by hospitalization and amputation. To prevent (re)ulcerations, custom-made orthopedic shoes are considered essential. However, adherence to wearing the orthopedic shoes is low, and improving adherence was not successful in the past. We propose a novel care approach that combines motivational interviewing (MI) with a digital shoe-fitting procedure to improve adherence to orthopedic shoes. The aim of this trial is to assess the (cost-)effectiveness of this novel care approach compared to usual care (no MI and casting-based shoe-fitting) in promoting footwear adherence and ulcer prevention.

**Methods:**

The trial will include people with diabetes, with IWGDF Risk categories 1–3, who have been prescribed orthopedic shoes. Participants will be randomized at the level of the podiatrist to the novel care approach or usual care. The primary outcome is the proportion of participants who adhere to the use of their orthopedic shoes, that is, who take at least 80% of their total daily steps with orthopedic shoes. A temperature microsensor will be built into the participants’ orthopedic shoes to measure wearing time continuously over 12 months. In addition, daily activity will be measured periodically using log data with an activity monitor. Data from the temperature microsensor and activity monitor will be combined to calculate adherence. (Re-)experienced complications after receiving orthopedic shoes will be registered. Questionnaires and interviews will measure the experiences of participants regarding orthopedic shoes, experiences of podiatrists regarding motivational interviewing, care consumption, and quality of life. Differences in costs and quality of life will be determined in a cost-effectiveness analysis.

**Discussion:**

This trial will generate novel insights into the socio-economic and well-being impact and the clinical effectiveness of the novel care approach on adherence to wearing orthopedic shoes.

**Trial registration:**

Netherlands Trial Register NL7710. Registered on 6 May 2019

## Administrative information

Note: the numbers in curly brackets in this protocol refer to SPIRIT checklist item numbers. The order of the items has been modified to group similar items (see http://www.equator-network.org/reporting-guidelines/spirit-2013-statement-defining-standard-protocol-items-for-clinical-trials/).

**Table:** 

Title {1}	Using motivational interviewing combined with digital shoe-fitting to improve adherence to wearing orthopedic shoes in people with diabetes at risk of foot ulceration: study protocol for a cluster-randomized controlled trial
Trial registration {2a and 2B}	Netherlands Trial Register, registration number NL7710
Protocol version {3}	Version 1, 22 January 2019
Funding {4}	This trial is funded by ZonMw (the Netherlands Organization for health Research and Development)
Author details {5a}	M. Jongebloed-Westra*, Department of Psychology, Health and Technology, Centre for eHealth Research and Wellbeing, TechMed Centre, University of Twente, PO Box 217, 7500 AE, Enschede, The Netherlands **C. Bode**, Department of Psychology, Health and Technology, Centre for eHealth Research and Wellbeing, TechMed Centre, University of Twente, PO Box 217, 7500 AE, Enschede, The Netherlands **J.J. van Netten**, Department of Rehabilitation, Amsterdam UMC, location Academic Medical Centre, University of Amsterdam, Amsterdam Movement Sciences, Meibergdreef 9, 1105 AZ, Amsterdam, The Netherlands; Diabetic Foot Unit, Department of Surgery, Hospital Group Twente, PO Box 7600, 7600 SZ, Almelo The Netherlands **P.M. ten Klooster**, Department of Psychology, Health and Technology, Centre for eHealth Research and Wellbeing, TechMed Centre, University of Twente, PO Box 217, 7500 AE, Enschede, The Netherlands **S.H. Exterkate**, Voetencentrum Wender, Sabina Klinkhamerweg 10, 7555 SK, Hengelo, The Netherlands; Voetmax Orthopedie, Sabina Klinkhamerweg 10, 7555 SK, Hengelo, The Netherlands **H. Koffijberg**, Department of Health Technology and Services Research, TechMed Centre, University of Twente, PO Box 217, 7500 AE, Enschede, The Netherlands **J.E.W.C. van Gemert-Pijnen**, Department of Psychology, Health and Technology, Centre for eHealth Research and Wellbeing, TechMed Centre, University of Twente, PO Box 217, 7500 AE, Enschede, The Netherlands *Corresponding author
Name and contact information for the trial sponsor {5b}	University of Twente, Enschede, The Netherlands
Role of sponsor {5c}	The funder had no influence in the study design; the collection, management, analysis, and interpretation of the data; writing of the report; and the decision to submit the report for publication, and had no ultimate authority over any of these activities.

## Introduction

### Background and rationale {6a}

Diabetes mellitus is one of the most common chronic diseases worldwide. The disease currently affects 425 million adults worldwide [1], and this number is expected to increase to 600 million people by 2035, due to population growth and aging [2]. A significant number of people with diabetes have foot ulcers (lifetime prevalence of 19–34%) leading to foot infection, amputation, and hospitalization [3]; immobility; and reduced quality of life [4]. In addition, diabetic foot ulcers account for high costs due to unemployment (loss of productivity) and social isolation, healthcare-related costs due to treatment, hospital admissions, and home care [3, 5–9]. Therefore, prevention of foot ulcers has high priority [3–9].

Early detection of risks, self-management, and protective footwear such as orthopedic shoes are considered essential to prevent re-ulceration [10, 11]. Adherence is crucial because patients who adhere to these strategies have significantly better outcomes than those who do not [12]. However, a randomized trial in The Netherlands found that adherence to orthopedic shoes is rather low, with only 46–49% of patients wearing their orthopedic shoes for at least 80% of daily total steps [13, 14]. Research into interventions to improve this adherence is scarce [15], but the explorative study by Keukenkamp et al. [11] showed that motivational interviewing (MI) has short-term positive effects on adherence. Motivational interviewing increased adherence to orthopedic shoes at home after 3 months from 31% (without MI) to 40% (with MI) indicating the beneficial consequences of this communication method [11]. Well-powered high-quality randomized trials are needed to better inform clinical practice about different methods to improve adherence to wearing orthopedic shoes [11, 12, 16].

The role of people’s motives and reasons for (not) adhere to wearing orthopedic shoes is largely unknown [10, 14, 16, 17] and has not been studied systematically [10, 13, 14]. Waaijman et al. [14] demonstrated some predictive value of lower BMI, severe foot deformity, and more appealing orthopedic shoes on adherence. However, their multivariate prediction model explained only 18% of the variance in adherence. This means that optimizing these predicting factors may have a limited effect on adherence and that other factors have to be taken into consideration for improving adherence substantially. Similar to the study of Waaijman et al. [14], most of the studies on diabetic footwear focused on patients’ physical and clinical characteristics rather than social and psychological factors. Until now, the patient perspective on wearing orthopedic shoes, possible psychological barriers, and living and working environments were neglected in adherence studies. However, clinical practice shows that focusing only on clinical aspects (re-ulcerations) and the quality of orthopedic shoes is not enough to improve adherence to orthopedic shoes: wearing orthopedic shoes also requires intrinsic motivation [18].

To improve motivation and adherence, various authors have recommended a combination of improved education and communication with better-fitting orthopedic shoes [10, 11]. First, an observational study found that higher patient satisfaction with the communication between patients and caregivers was associated with increased long-term use of orthopedic shoes [19]. We believe that such a working alliance can be created via motivational interviewing since “motivational interviewing is a collaborative, goal-oriented style of communication with particular attention to the language of change. It is designed to strengthen personal motivation for and commitment to a specific goal by eliciting and exploring the person’s own reasons for change within an atmosphere of acceptance and compassion” [20]. Keukenkamp et al. concluded that motivational interviewing is a promising method for the given purpose and patient group [11]. However, motivational interviewing requires the caregiver to engage with the patient as an equal partner and to not give unsolicited advice or direct, confront, warn, or instruct the patient. Motivational interviewing requires discipline and self-awareness from the caregiver, and mastering motivational interviewing takes practice and time [21]. Podiatrists work at the front lines of diabetic foot care and work with high-risk diabetic patients and are motivated to help guide patients toward better self-care. However, they do not necessarily have the skills to do so effectively. Gabbay et al. believe that there is a great opportunity for podiatrists to explore motivational interviewing to change patient behavior [22]. This suggests that the shoe-fitting procedure plays an important role in creating a working alliance between patient and podiatrist to increase acceptance of and adherence with orthopedic shoes by shared decision-making embedded in person-centered communication [23].

A second factor to increase adherence may be a better fit of the orthopedic shoes. Although perceived orthopedic shoe comfort was not found to be a predictor of adherence for people with diabetes in a previous study [14], van Netten et al. [24] found that all aspects of usability are relevant in relation to the use of orthopedic shoes in people with different pathologies. Therefore, the fit of orthopedic shoes will likely affect the adherence of people with diabetes in practice. Currently, orthopedic shoes are mostly produced using a solid 3D mold known as a “shoe last” [25]. These lasts are traditionally made using casting-based methods. However, casting methods are expensive, time-consuming, and complicated due to constraints imposed by manual measurements of several foot dimensions and manual crafting (trial-and-error) of the shoe last to fit the patient’s foot dimensions [26, 27]. A digital shoe-fitting procedure, using a high-end 3D scanner to scan the foot instead of creating a mold around the foot, might be more accurate, patient-friendly, and time-efficient. In this method, the digital scan of the foot is modeled into a patient-specific last that can be milled by a last-milling machine. Although slowly implemented in clinical practice, improvements in scanning methods are expected to lead to a better fit of the orthopedic shoes and therefore to improve adherence to wearing orthopedic shoes.

The factors reviewed above suggest that a multidisciplinary and biopsychosocial approach can help to improve adherence to wearing orthopedic shoes [28] since different aspects have to be taken into account simultaneously to improve adherence meaningfully. However, there is little knowledge about the effectiveness of interventions, and the cost-effectiveness of the novel care approach (motivational interviewing combined with digital shoe-fitting) has not been studied at all [29]. Therefore, the aim of the current study is to assess the (cost-)effectiveness of this novel care approach compared to usual care (no motivational support and casting-based shoe-fitting) in improving adherence to wearing orthopedic shoes and ulcer prevention. This study will generate insights into the socio-economic impact of the novel care approach on adherence to orthopedic shoes. These are crucial steps toward better ulcer prevention in high-risk people with diabetes and to improve their quality of life.

### Objectives {7}

The primary objective is to compare the proportion of participants who sufficiently adhere to using their orthopedic shoes (that is, who take at least 80% of their total daily steps with orthopedic shoes) between participants receiving the novel care approach which consists of motivational interviewing combined with a new digital shoe-fitting procedure and participants receiving usual care.

The secondary objectives are to compare between the novel care approach and usual care: 1) the level of adherence to the use of orthopedic shoes; 2) change in adherence; 3) total wearing time; 4) the proportion of participants (re-)experiencing complications during 1 year follow-up; 5) the participant-perceived quality of life; and 6) the experiences of participants regarding their knowledge about the aim of orthopedic shoes, their satisfaction with communication with the pedorthist regarding wearing orthopedic shoes, their intentions to change wearing behavior, and their satisfaction with orthopedic shoes; in addition, 7) to determine the experiences of podiatrists regarding their knowledge about MI and their experiences and attitudes toward applying MI in this group of patients; 8) the differences in the application of MI between the MI-trained and non-MI-trained podiatrists; and 9) to calculate the differences in costs between the novel care approach and usual car and to assess 10) the cost-effectiveness of the novel care approach compared with usual care.

### Trial design {8}

A multicenter, cluster-randomized controlled trial with (cost-)effectiveness analysis and qualitative and quantitative process analyses.

## Methods: participants, intervention, and outcomes

### Study setting {9}

People with diabetes treated by a pedorthist of Voetmax Orthopedie, for whom foot care is reimbursed in the Dutch healthcare system, will be recruited at different locations of Voetencentrum Wender and Voetmax Orthopedie, located in the east of The Netherlands. Randomization will be performed at the level of the podiatrists (see the “Sequence generation {16a}” section).

### Eligibility criteria {10}

In order to be eligible to participate in this study, a participant must meet all of the following inclusion criteria:

- A clinical diagnosis of diabetes mellitus type 1 or 2
- Aged 18 years or older
- With or without previous callus
- With or without previous ulcers
- Identified with risk profiles 2, 3, or 4, according to the “zorgmodule preventie diabetische voetulcera 2014” [30]. Internationally better known as the IWGDF Risk 1–3 [31], see Table 1

**Table 1:** Care profiles 2–4 versus IWGDF Risks 1–3 for eligible patients

Care profile	Category	Ulcer risk	Characteristics
2	1	Moderate	LOPS + PAD
3	2	Moderate	LOPS + foot deformity
			PAD + foot deformity
4	3	High	LOPS or PAD, and one or more of the following: - History of a foot ulcer - A lower-extremity amputation - End-stage renal disease

*LOPS* loss of protective sensation, *PAD* peripheral artery disease
- Eligible for a prescription of orthopedic shoes

Participants will be excluded when they meet any of the following exclusion criteria:

- Did not receive orthopedic shoes, but instead an adaption to confection shoes or semi-orthopedic shoes
- Have a foot ulcer
- Active Charcot’s neuro-arthropathy
- Have a foot infection
- Unable to walk
- Unable to read and understand the study instructions

### Who will take informed consent? {26a}

During a multidisciplinary consultation with the pedorthist and medical specialist, the patient will be asked if he/she decided to participate in this study. If he/she decided to participate, the investigator or the investigator’s representative will ask the patient to sign an informed consent.

All podiatrists will provide written informed consent for contribution to the study. They will be asked by the coordinating investigator.

### Additional consent provisions for collection and use of participant data and biological specimens {26b}

On the informed consent form, participants will be asked if they agree to the storage and use of their personal information for future research on adherence to orthopedic shoes. By signing the informed consent form, participants give permission to inform their podiatrist and pedorthist about their participation in the study and inform them when there are unexpected findings that are or could be important to the health of the participant, record one of the consultations with their podiatrist, for the research team to request medical information from their medical files, and when necessary to share data with the competent authorities. Biological specimens will not be collected for this trial.

### Interventions

#### Explanation for the choice of comparators {6b}

The novel care approach will be compared to the usual care as this is a standard clinical practice in The Netherlands.

#### Intervention description {11a}

##### Novel care approach

**Fig. 1:**
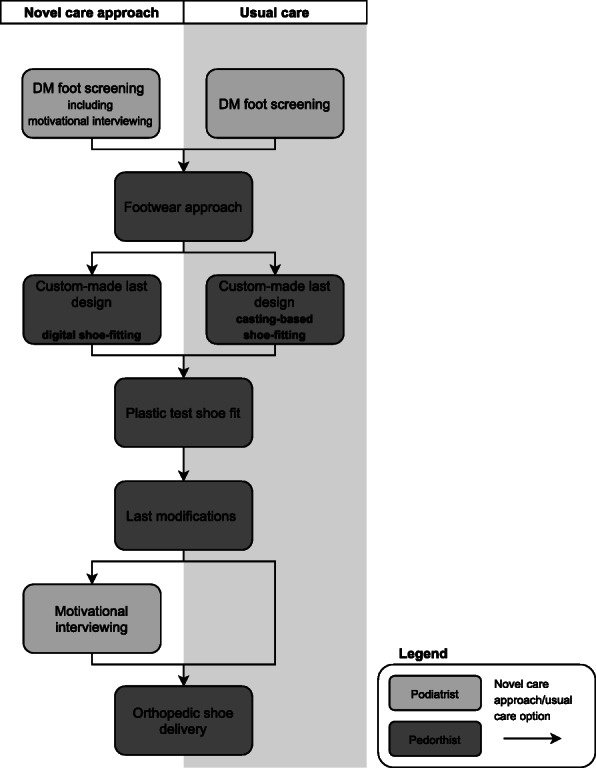
Novel care approach versus usual care

Participants will receive a combination of MI by the podiatrist to improve acceptance of orthopedic shoes and adherence, and a new digital shoe-fitting procedure by the pedorthist. When the participant needs orthopedic shoes, the podiatrist will refer the participant to a pedorthist to measure for orthopedic footwear (see Fig. 1 for further details).

The new shoe-fitting procedure will consist of using a digital iPad scanner with a scan frame where the foot will be scanned (half-)weight bearing. A calibrated length is used to scale the scan results to absolute dimensions.

##### Usual care

MI is currently not provided in standard clinical practice in The Netherlands. Usual care will consist of foot care from the podiatrist. When the participant needs custom-made shoes, the podiatrist will refer the participant to a pedorthist to measure for orthopedic footwear by a casting-based shoe-fitting procedure [32–35] (see Fig. 1 for further details).

##### Motivational interviewing training of podiatrist

Motivational interviewing entails a number of general coaching principles, such as avoiding argumentation and direct confrontation, but rolling with the existing reservations and supporting self-efficacy, optimism, and behavioral intentions in patients to support the change of behavior with regard to wearing orthopedic shoes [20]. A certified MI trainer (Motivational Interviewing Network of Trainers (MINT)) will be training the podiatrists in MI during a 3-day basic training. The podiatrists will be trained to incorporate the specific coaching and communication techniques of MI in their consultation hours with the aim to increase adherence to wearing orthopedic shoes in our target group.

### Criteria for discontinuing or modifying allocated interventions {11b}

Participants can leave the study at any time for any reason if they wish to do so without any consequences. The investigator can also decide to withdraw a participant from the study for urgent medical reasons. The outcomes will no longer be collected, and participants’ data that have been collected up to that moment will be included in the analysis. If participants drop out of the study, additional participants will be included until *N* = 220.

Given the low risk of the intervention, there are no criteria set for premature termination of the study, because this is not to be expected.

### Strategies to improve adherence to interventions {11c}

All participants will be contacted by the coordinating investigator before all their consultations during the study to remind them about the consultation and to bring their orthopedic shoes equipped with a temperature microsensor. If they received questionnaires and/or an activity monitor during the consultation, and this is not returned in 2 weeks after the consult, the participant will be contacted by the coordinating investigator to fill in the questionnaires and return them and/or return the activity monitor.

### Relevant concomitant care permitted or prohibited during the trial {11d}

All participants are allowed to receive any form of (foot) care that they need, e.g,. regular appointments with a podiatrist and/or diabetes pedicure, if necessary wound treatment at a multidisciplinary diabetic foot clinic, and regular appointments with the a pedorthist regarding their orthopedic shoes. This (foot) care will be measured with the iMCQ (Institute for Medical Technology Assessment (iMTA) Medical Consumption Questionnaire).

### Provisions for post-trial care {30}

The multicenter sites have a liability insurance which is in accordance with article 7 of the WMO [36]. This insurance provides cover for damage to research subjects through injury or death caused by the study. The insurance applies to the damage that becomes apparent during the study or within 4 years after the end of the study. There are no other provisions for post-trial care.

### Outcomes {12}

The primary outcome is the proportion of participants who adhere to wearing their orthopedic shoes (see the “Proportion participants being adherent” section). We define adherence as minimally 80% of daily steps taken with orthopedic shoes based on the data of a randomized trial in The Netherlands [13, 14].

Secondary outcomes are 1) the level of adherence to wearing orthopedic shoes during 1 week at 3 and 6 months after inclusion; 2) the change in adherence between 3 and 6 months after inclusion; 3) total wearing time during 1 year follow-up; 4) the proportion of participants (re-)experiencing complications during 1 year follow-up; 5) the participant-perceived quality of life at inclusion and 3 and 6 months after inclusion; 6) the experiences of participants regarding their knowledge about the aim of orthopedic shoes, their satisfaction with communication with the pedorthist regarding wearing orthopedic shoes, their behavioral intentions, and their satisfaction with orthopedic shoes, at inclusion and 6 month after inclusion; 7) the experiences of podiatrists regarding their knowledge about motivational interviewing and their experiences and attitudes toward applying motivational interviewing in this group of patients, after all participants are included; 8) the application of motivational interviewing; and 9) foot care-related costs during 1 year follow-up. For an overview of all time points, see Table 2 (see the “Participant timeline {13}” section).

**Table 2:**
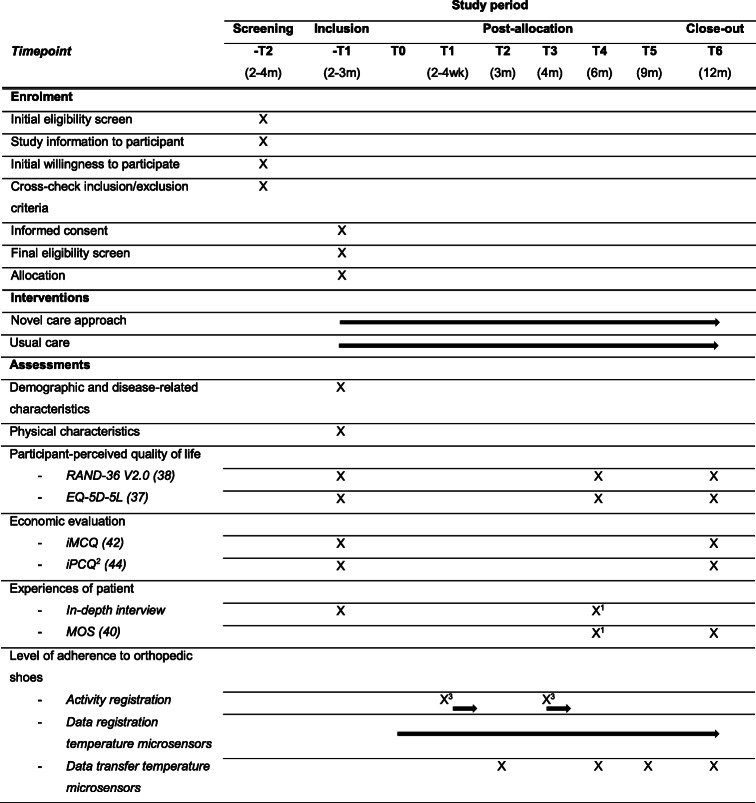
Overview of the measurements of the study parameters during the study

*Abbreviations*: *EQ-5D-5L* 5-Level EuroQol Quality of Life Scale, *iMCQ* iMTA Medical Consumption Questionnaire, *iPCQ* iMTA Productivity Cost Questionnaire, *m* months, *MOS* Monitor Orthopedic Shoes post-part, *RAND-36 V2.0* Research and Development 36-item Health Survey version 2.0, *wk* weeks

^1^The participants, who will not be approached for the in-depth interview, will be asked to fill in MOS

^2^The iPCQ will also be taken from participants after (re-) experiencing complications; taken 4 weeks after the complication was diagnosed

^3^Activity registration during 1 week

[37] [38] [40] [42] [44] and [47]

#### Proportion participants being adherent

The main study parameter is the proportion of participants who adhere to wearing their orthopedic shoes, defined as minimally 80% of steps taken with orthopedic shoes. The level of adherence (see the “Level of adherence to orthopedic shoes” section) will be based on log data from temperature microsensors in the orthopedic shoes and data from the activity monitors provided to all participants over two 1-week periods measured at time point “T1” and time point “T3” (Table 2). The proportion of adherent participants will be objectively determined based on the combined level of adherence of the two measurements (the mean of time point “T1” and time-point “T3”).

#### Level of adherence to orthopedic shoes

The level of adherence to the use of orthopedic shoes will be determined by the percentage of total steps during the full recording period that the orthopedic shoes were worn and will be calculated as follows:

$$ \mathrm{Adherence}=\frac{\Sigma \kern0.5em \mathrm{steps}\ \mathrm{wearing}\ \mathrm{orthopaedic}\ \mathrm{shoes}}{\Sigma \kern0.5em \mathrm{steps}}\times 100 $$

Total steps wearing orthopedic shoes will be based on log data from temperature microsensors in the orthopedic shoes of all participants, and total steps will be based on using the data from the activity monitors over two times 1 week period measured at time point “T1” and time point “T3” (Table 2).

Adherence to wearing orthopedic shoes and daily step count will be assessed using raw data from the temperature microsensors using and processed by MATLAB (The MathWorks Inc., Natick, MA, USA). Participants will be included in the analyses only if at least four complete days of recording, including one weekend day, are available [14]. When both the temperature microsensor and the step activity monitor show activity during recording, it will be assumed that the participant walked with the orthopedic shoes. If only step activity is recorded, it will be assumed that the participant was walking barefoot or walking in non-prescribed shoes.

#### Change in adherence

The change in adherence will be determined by the mean of the level of adherence to the use of orthopedic shoes measured 6 months after inclusion minus the mean of the level of adherence to the use of orthopedic shoes measured 3 months after inclusion.

#### Total wearing time

The total wearing time of the orthopedic shoes during the 12-month follow-up will be based on log data from temperature microsensors in the orthopedic shoes of all participants and will be analyzed for different periods.

#### Complications

The proportion of participants (re-)experiencing complications (i.e., one or more ulcers or abundant callus that requires debridement, not present at baseline, or lower-extremity amputation) will be determined by the registration of (re-)experienced complications after receiving their orthopedic shoes, up to 1 year after baseline. All complications will be registered and photographed by podiatrists, who are informed by the participant if complications occur (in > 95% of complications cases, the podiatrist is the first to hear from the participants). If it is necessary to obtain details on specific complications, general practitioners or orthopedic surgeons will be contacted. Photographs will be assessed by observers blinded to treatment allocation to confirm the type and/or severity of the complication.

#### Participant-perceived quality of life

The participant-perceived quality of life of participants will be assessed with the 5-Level EuroQol Quality of Life Scale (EQ-5D-5L) questionnaire [37] and RAND-36 item Health Survey V2.0 (RAND-36 V2.0) [38]. The negative impact of complications on quality of life will be based on the literature. Quality-adjusted life years (QALYs) will be calculated based on the quality of life calculated from the EQ-5D-5L and the time duration between measurements, or the time until the end of life, based on the Dutch tariff established for the EQ-5D-5L [39].

#### Experiences of participant

A mixed methods approach will be applied to obtain the participants’ experiences and perspectives regarding their knowledge about the aim of orthopedic shoes, their satisfaction with communication with the pedorthist regarding wearing orthopedic shoes, their intentions to change wearing behavior, and their satisfaction with orthopedic shoes. A quantitative questionnaire (Monitor Orthopedic Shoes post-part (MOS) [40]) will be used to measure participant experiences on orthopedic shoes, use, and usability at 6 and 12 months after baseline. The information from the MOS will be complemented with data from in-depth interviews with 30 participants at baseline and 6 months after baseline. Participants will be selected randomly for the interviews: 15 participants of the intervention group and 15 of the control group.

#### Experiences of podiatrist

A mixed methods approach will be applied to obtain the MI-trained podiatrist experiences, with quantitative analysis of observed application of MI by the MI-trained podiatrist scored with the Motivational Interviewing Treatment Integrity (MITI) [41] and with interview results. The in-depth interviews will be taken after the last participant had his/her last consultation with the podiatrist.

#### Application of motivational interviewing

Between 1 and 2 months after the MI training, all podiatrists (the MI-trained and the non-MI-trained) will audio record some conversations with the participants for the assessment of applying MI or not. A health psychologist, educated in training motivational interviewing by the MINT, will be responsible for scoring the quality of the MI applied by the podiatrist with the MITI. To explore whether there is, as expected, a difference between the MI-trained podiatrists and the non-MI-trained podiatrists in the application of MI principles, also conversations from the non-MI-trained podiatrists will be scored with the MITI.

#### Foot care-related costs

Healthcare resource use of participants will be determined using the Institute for Medical Technology Assessment (iMTA) Medical Consumption Questionnaire (iMCQ) [42]. Cost prices will be calculated according to the 2015 Dutch guideline for health economic evaluation [43]. If relevant, costs of medication use will be derived from the Dutch formulary increased with a pharmacist’s charge. Costs of diagnostic tests will be based on Dutch tariffs, and, if applicable, costs of over-the-counter medication and alternative medicines will be based on average retail prices. Costs of consulting a general practitioner or medical specialist or other procedures and hospitalizations will be based on the 2015 Dutch guideline for health economic evaluation [31] or charges if no other estimates are available. The potential productivity losses from complications of the foot or the orthopedic shoes will be assessed using the iMTA Productivity Cost Questionnaire (iPCQ) [44] instrument among all participants at baseline and 12 months after baseline, and additionally among participants who present with complications, at 4 weeks after the complication was diagnosed [32]. A friction cost approach will be applied to estimate the productivity losses as defined in the Dutch costing manual and based on the reference costs of not being able to perform paid or unpaid work.

### Participant timeline {13}

An overview of the study design and the main procedures that participants will undergo during the course of the study are shown in Table 2. The participant will receive the novel care approach or usual care. All standardized instruments used in the study procedure are described in Table 2.

The participants will be followed from inclusion up to 12 months after receiving their orthopedic shoes, with visits planned at different moments during this period for consultations with the podiatrist, pedorthist, and investigator. During every study consultation with the investigator, the participant will be asked about complications.

#### Time point “− T2”—screening

Eligible patients, who will be referred to the pedorthist for orthopedic shoes, will be informed about the study by the podiatrist and will receive the information brochure and informed consent form. The podiatrist will ask permission to send contact details to the research team. On receipt of that permission, the podiatrist will provide details of the patient to the coordinating investigator. The coordinating investigator will contact the patient in order to further explain the study and answer any questions the patient may have. After this contact, the patient will be given a minimal 1 week to decide to participate in this study.

#### Time point “− T1”—inclusion

After referral of the podiatrist, the pedorthist and medical specialist will decide together, during a multidisciplinary consultation, which type of shoes the patient will need. When instead of custom-made orthopedic shoes convection shoes or semi-orthopedic shoes will be prescribed, the patient cannot be included in the study. After the patient has been prescribed custom-made orthopedic shoes, he/she will be asked if he/she decided to participate in this study, and the investigator or the investigator’s representative will ask the patient to sign informed consent. Also, the demographic data (age, gender, ethnicity, height, and weight), diabetes type and duration, risk profile, ambulatory status, history regarding the use of orthopedic shoes, educational status, socioeconomic status, and capacity for self-care are completed. Subsequently, data on the presence of peripheral artery disease, peripheral neuropathy, foot deformities, and history of previous foot ulceration and amputation will be recorded, and the participants will be asked to fill in specific questionnaires (time point “− T1” at Table 2).

Thereafter, during a consult with the pedorthist, the orthopedic shoes will be fitted. The pedorthist will provide the new digital shoe-fitting procedure or the casting-based procedure depending on whether the podiatrist is trained in MI or not.

Within 1 to 6 weeks, the participant from the intervention group will have another consultation with the MI-trained podiatrist (first consult: participant was referred to the pedorthist). The podiatrist will apply MI in this conversation. From all included participants, thirty participants, 15 from both groups, will be approached for an in-depth interview about their perspective on and experiences with orthopedic shoes before receiving these shoes. This interview will be performed by one of the investigators.

#### Time point “T0”—receiving the first pair of orthopedic shoes (baseline)

Two to 3 months after the multidisciplinary consultation, the participant will receive their first pair of orthopedic shoes during a consultation with the pedorthist. A temperature microsensor (Orthotimer®) is embedded in the insole of the orthopedic shoes for determining adherence by measuring and recording wearing time.

#### Time point “T1”—shoe control after receiving the first pair of orthopedic shoes

The participants will have another consultation with the pedorthist (2 to 4 weeks later) for shoe control and fitting the second pair of orthopedic shoes. During this consultation, they receive an activity monitor and instructions from the investigator. The participants will be instructed to wear the activity monitor (Misfit Shine 2™) for a whole week starting the day after this consultation (24 h per day).

Six months after the first consultation with the podiatrist, most participants will have another regular consultation with the podiatrist for foot care. If the podiatrist is MI-trained also in this consult, MI will be applied.

#### Time point “T2”—receiving the second pair of orthopedic shoes

To deliver the second pair of orthopedic shoes, a regular consultation will be made with the pedorthist 3 months after receiving the first pair of shoes. The second pair of shoes will also be provided with a temperature microsensor (Orthotimer® microsensor) embedded in the insole of the orthopedic shoes. During this consult, the temperature microsensor of the first pair of shoes will be read out with the reading device (Orthotimer® readerdevice) by the investigator.

#### Time point “T3”—shoe control after receiving the second pair of orthopedic shoes

Two to 4 weeks after receiving the second pair of orthopedic shoes, another regular control consultation will be planned. Again, the patients will receive an activity monitor to register their activities. The activity monitor (Misfit Shine 2™) will also be worn again for one whole week (24 h per day).

#### Time point “T4”—consultation with the investigator

Three months after receiving the second pair of orthopedic shoes, a consultation with the investigator will be made to read out the temperature microsensors of both pairs of shoes. The participants will also be asked to fill in some questionnaires (time point “T4” Table 2). The same 30 participants as before will be approached for a second in-depth interview about their perspective about and experiences with orthopedic shoes, and the other participants will be asked to fill in the MOS instead.

#### Time point “T5”—consultation with the podiatrist

One year after the first consultation with the podiatrist, every participant will have a regular consultation with the podiatrist for control of their feet. During this consult, the temperature microsensors of both pairs of shoes will be read out by the investigator. And also as before, in the consultations with a MI-trained podiatrist, MI will be applied.

#### Time point “T6”—close out

A last consult with the investigator will be planned about 6 months after receiving the second pair of orthopedic shoes to read the temperature microsensors out both pairs of shoes and to fill in some questionnaires (time point “T6” Table 2).

### Sample size {14}

In this study, 220 eligible participants will be required (110 in both arms), accounting for potential dropouts. Given that MI has been found to increase adherence to orthopedic shoes at home after 3 months from 31% (without MI) to 40% (with MI) [11], we conservatively anticipate that the MI provided by the podiatrists will improve adherence by at least 10%. Moreover, we estimate the use of a digital shoe-fitting procedure by the pedorthist rather than a casting-based shoe-fitting procedure to increase adherence with at least another 10%, due to the experienced improvement of last accuracy and orthopedic shoe-fitting.

Based on the observed 3 months adherence of 59% for the usual care procedure [11], we expect the 1-year overall adherence to drop to 40% for the usual care, and to be 40% + 10% + 10% = 60% for the novel care approach including the MI. Adherence often decreases over a longer term as shown in the study of Keukenkamp et al.: over time the improved adherence returned to baseline levels [11].

The sample size calculation is performed using the “clusterPower” package in R, based on a two-sided alpha of 0.05, power of 0.80, and intraclass correlation of 0.01 of patients within podiatrists. This demonstrated that this effect in a generalized linear mixed model would require 200 participants in total. Recognizing loss to follow-up, which occurred in 6 + 4 = 10 out of 85 + 86 = 171 participants in a recent study in this context [13], that is ~ 6%, we conservatively aim to include 220 participants in total.

### Recruitment {15}

Eligible patients, who will be referred to the pedorthist for orthopedic shoes, will be informed about the study by the podiatrist and will receive the information brochure and informed consent form. The podiatrist will ask permission to send contact details to the research team. On receipt of that permission, the podiatrist will provide details of the patient to the coordinating investigator. The coordinating investigator will contact the patient in order to further explain the study and answer any questions the patient may have. During a multidisciplinary consultation with a pedorthist and a medical specialist, the patient will be asked if he/she decided to participate in this study. If he/she decided to participate, the investigator or the investigator’s representative will ask the patient to sign informed consent.

### Assignment of interventions: allocation

#### Sequence generation {16a}

Randomization will be performed at the level of the podiatrists. Based on their working location (distance from Voetmax Orthopedie locations), working calendar, working days, and number of patients with diabetes, we included 20 podiatrists of Voetencentrum Wender in this study. Since the podiatrists still differ widely in their number of patients seen and experience with the specific target group, stratified randomization will be used for the group of podiatrists. Four of the 20 podiatrists run special consultations for people with diabetes and are therefore likely more specialized in diabetic foot disease. These four podiatrists are split into two groups, based on the number of patients seen per year (based on the figures of 2019), and equally randomized to the group who receive MI training or to the group who do not receive MI training. The other 16 podiatrists are randomized next, also stratified by the number of patients seen per year (based on four strata using last year’s figures). The randomization is done centrally by an independent researcher using www.sealedenvelope.com.

Each podiatrist will exclusively provide either the MI intervention or usual care. Thereafter, the pedorthist will provide the new digital shoe-fitting procedure for the intervention group of participants or the casting-based shoe-fitting procedure for the control group.

#### Concealment mechanism {16b}

The participants have not randomized themselves, because the background assignment of the treating podiatrist (being trained in MI or not) will determine the treatment allocation of the participants. Randomization is performed at the level of the podiatrists to avoid contamination between intervention and control participants. Therefore, the randomization sequence will not be concealed from the podiatrists. Because each podiatrist will exclusively provide either the MI intervention or usual care, and thereafter, the pedorthist will provide the new digital shoe-fitting procedure for the intervention group of participants or the casting-based shoe-fitting procedure for the control group, and the randomization sequence will also not be concealed from the pedorthists.

All participant data is pseudonymized, but because the investigators have access to the coding of the personal data of the participants, the randomization sequence will also not be concealed from them.

#### Implementation {16c}

Not applicable, because participants will not be randomized. Randomization will be performed at the level of the podiatrists (see the “Sequence generation {16a}” section).

### Assignment of interventions: blinding

#### Who will be blinded {17a}

Blinding and concealed treatment allocation for podiatrists is not feasible, because of the way of randomization (see the “Concealment mechanism {16b}” section). Outcome assessments and analyses are not performed by independent staff, but by the investigators themselves, and therefore, they are also not blinded to the treatment allocation (see the “Concealment mechanism {16b}” section).

#### Procedure for unblinding if needed {17b}

Not applicable, because this is an open-label trial and because the outcome assessments and analyses are not performed by independent staff (see the “Concealment mechanism {16b}” section).

### Data collection and management

#### Plans for assessment and collection of outcomes {18a}

All standardized instruments used in the study procedure are described in Table 2. Information on the other study instruments can be found below.

##### Orthotimer® and reader device

The Orthotimer® microsensor (Rollerwerk Medical Engineering & Consulting, Balingen, Germany) will be used for continuous, long-term measurement of adherence and is a valid sensor to measure temperature in footwear [45]. The microsensor measures the temperature within the footwear every 15 min (96 measurements per day) and stores these data for 100 days before overwriting the oldest data. Longer observation periods will be possible by reading out the temperature microsensor data before this deadline. Every temperature microsensor reading will be stored with a date- and timestamp. In case participants will be prescribed more than one pair of orthopedic shoes, in both pairs of shoes, a temperature microsensor will be placed and data from both temperature microsensors will be combined.

The temperature microsensor is controlled with the wireless reading device and the saved wearing time dates are transferred to the respective software. The reading device can be connected with the computer via a USB plug. The software is used to control the temperature microsensor as well as to perform the wearing time analysis of the participant data.

##### Activity monitor

The Misfit Shine 2™ (Misfit Wearable, Burlingame, CA, USA) is a small tri-axial accelerometer which will be carried at the lower extremity. The Misfit Shine 2™ measures steps, calories burned, distance, activity types, sleep quality, and duration. The Shine 2 holds up to 30 days of activity data. The reliability of the Misfit shine is good [46]. Data can be transferred reliably and wireless to the Health app (iPhone) or Google Fit (Android phone), which will be connected to the TIIM-app (BMS Lab, University of Twente), so the data will be collected at a secured server.

##### Interview structure

The interviews will contain open-ended and closed questions and will be structured according to the relevant concepts for adherence to orthopedic shoes. To gain insight into the perspective of participants, motivations for and experienced advantages and difficulties regarding frequency, proper fit, and adequate wearing of orthopedic shoes will be discussed with the participants.

To examine the experiences of MI-trained podiatrists, the following topics will be discussed in the interview: knowledge, adoption and implementation of the motivational interviewing procedure among podiatrist, and their experiences and attitudes toward applying MI in this group of participants.

#### Plans to promote participant retention and complete follow-up {18b}

The patients will receive extensive information about the study setup and requirements during the recruitment. Once in the study, to promote complete follow-up, all participants receive a phone call before all their consultations during the study to remind them about the consultation (see the “Strategies to improve adherence to interventions {11c}” section).

#### Data management {19}

As required by the funder (ZonMw), a data management plan has been developed for this study. The participants will be coded by the letter of the participating center (one letter) and the letter of intervention or control group (one letter) followed by the number of the participant (four digits). All personally identifiable information will be saved in a locked cupboard with the coordinating investigator and on a computer protected with a password. All data will be collected on paper and then entered electronically in an Excel database by the investigator. All pseudonymized study data will be entered in a specific server facility (LISA) of the University of Twente for storage and archiving. The handling of the data will comply with the EU General Data Protection Regulation and the Dutch Act on Implementation of the General Data Protection Regulation (Uitvoeringswet AVG, UAVG). All study information will be saved for 10 years after the study ends in DANS (Data Archiving and Network Services). Access to original data on paper will be kept in a locked cupboard at the university with the coordinating investigator during the study.

#### Confidentiality {27}

All collected data will be pseudonymized by the coordinating investigator. The key code will be stored on a different secured server than the data and will be password protected. The principal investigator will decide who of the research group will have access to the data. Names of the participants will only be recorded on the informed consent form, which will be kept in a locked cupboard with the coordinating investigator, separated from the digital data and without a possibility to trace the data.

#### Plans for collection, laboratory evaluation, and storage of biological specimens for genetic or molecular analysis in this trial/future use {33}

Not applicable, because no samples will be collected.

## Statistical methods

### Statistical methods for primary and secondary outcomes {20a}

Statistical analysis and the cost-effectiveness analysis will be carried out with R environment for statistical computing (R Foundation, Vienna, Austria [47]). For statistical analyses, a significance level of *P* < 0.05 will be adopted.

#### Descriptive statistics

Anthropometric data, other participant characteristics, and data from adherence to orthopedic shoes and step count will be presented as mean or median with their standard deviation or the frequencies will be presented. Differences in the baseline characteristics between the participants receiving the novel care approach and usual care will be tested with a *t*-test, Mann-Whitney *U* test, chi-square test, or Fisher’s exact test, depending on the type of variables and being normally distributed or not.

#### Primary study outcome

Between-group differences in the proportion of participants who adhere to the use of their orthopedic shoes, that is, take at least 80% of their total steps with orthopedic shoes will be tested using a generalized linear mixed model (GLMM). A logistic link function will be used for the binary outcome on participant level (adherent yes/no) and random effects for podiatrists will be included.

#### Secondary study outcome

Differences in the level of adherence to the use of orthopedic shoes of participants and differences in the proportion of participants (re-)experiencing complications after receiving their orthopedic shoes, up to 1 year after baseline between the two groups of participants will also be tested using appropriate generalized linear mixed models. The quantitatively measured aspects of the participant experiences and the experiences of the MI-trained podiatrist will be tested using the same approach. The type of GLMM depends on the variable that will be tested in the model.

The qualitative (verbal) interview data of the experiences of the participant and the MI-trained podiatrist will be summarized with two code schemes (one for the participants’ experiences and one for the podiatrists’ experiences). The code schemes will be developed by combining inductive and deductive thematic analysis. Content and frequency of the main themes will be compared for the two groups of participants, and this information will be triangulated with the quantitative information on the experiences of participants to explain in more depth the results of adherence and in order to formulate implementation recommendations from the patients’ perspective. This triangulation approach will also be applied for the quantitative and qualitative data of the MI-trained podiatrists.

### Interim analyses {21b}

Not applicable, because no interim analyses are planned because there are no anticipated risks to participation in this study.

### Methods for additional analyses (e.g., subgroup analyses) {20b}

#### Cost-effectiveness analysis (CEA)

The cost-effectiveness of the novel care approach compared with usual care will be determined by dividing the difference in the mean costs (in Euros) by the difference in the mean health outcomes (in QALYs) to estimate the incremental cost-effectiveness ratio (ICER). For this trial-based, short-term cost-effectiveness analysis (CEA) with 1-year time horizon bootstrapping will be applied to determine the uncertainty in this ICER. The cost-effectiveness analysis for a lifetime time horizon will be model-based, using data from the literature as well as the trial data. Here, probabilistic sensitivity analysis will be applied to assess how uncertainty in model input parameters results in uncertainty in the ICER. The results will be presented in incremental cost-effectiveness planes and cost-effectiveness acceptability curves.

#### Methods in analysis to handle protocol non-adherence and any statistical methods to handle missing data {20c}

Between-group differences in the proportion of participants who adhere to the use of their orthopedic shoes, that is, who take at least 80% of their total daily steps with orthopedic shoes, will be tested in an intention-to-treat analysis using a generalized linear mixed model (GLMM) which can inherently deal with data missing at random.

### Plans to give access to the full protocol, participant level-data, and statistical code {31c}

The full protocol, pseudonymized dataset, and statistical code will be available on request after the results of the study have been published.

### Oversight and monitoring

#### Composition of the coordinating center and trial steering committee {5d}

This is a multicentered study designed, performed, and coordinated at the University of Twente, Voetmax Orthopedie, and Voetencentrum Wender. Support for the trial is provided by the following:

- Principal investigator: takes supervision of the trial.
- Coordinating investigator: preparation of protocol and revisions, ethics committee application, trial registration, visits the podiatrists and pedorthists during the start-up phase, organizes the MI-trainings given by MI-trainers to podiatrists, supports the logistics for patient accrual, take informed consents, monitors inclusion of patients, coordinates study visits, repeated measurements and collection of log data from sensors, organizes data acquisition, collection and storage, analyses and manages the primary and secondary outcome data, prepares the first draft of the manuscripts, and prepares progress reports for the project team/steering committee
- Project team/steering committee: design of the study, check study progress and approve protocol amendments and recommendations, and approve publication of study reports; meets monthly
- Study physicians (podiatrists/pedorthist): identify potential recruitments and take informed consent if possible
- Patient experience experts: advice project team during the inclusion period
- Advisory board: discuss the findings and implementation strategy with the project team and with the international network of the advisory board

#### Composition of the data monitoring committee, its role, and reporting structure {21a}

Because of the low burden and minimal risks, no data monitoring committee was appointed. The investigators are responsible for procedures of data monitoring.

#### Adverse event reporting and harms {22}

Adverse events are defined as any undesirable experience occurring to a participant during the study, whether or not considered related to the trial procedure. Because this trial was exempt from full medical ethical approval, all adverse events reported spontaneously by the participants or observed by the podiatrist, pedorthist, or the investigator or her staff will be registered by the investigator in the Excel database, and consequences will be discussed in the project team. As always, it is possible that problems may arise with the participant’s feet or orthopedic shoes, for which the participant will receive usual care performed by the podiatrist and/or pedorthist (see the “Relevant concomitant care permitted or prohibited during the trial {11d}” section).

#### Frequency and plans for auditing trial conduct {23}

To facilitate compliance with Good Clinical Practice guidelines, the investigator will permit study-related monitoring, audits, and inspections by authorized organizations. Aspects that will be monitored may include inclusion rate, informed consent progress, inclusion and exclusion criteria, trial master file, source data verification, safety reporting, trial procedures, and closing and reporting. Given the low risk of the intervention and because this trial was exempt from full medical ethical approval, extensive auditing is not considered necessary. Therefore, no audits are planned at this time as the principal investigator will be present to oversee all study activities as data are being collected.

#### Plans for communicating important protocol amendments to relevant parties (e.g., trial participants, ethical committees) {25}

Amendments are defined as changes made to the research. This trial was exempt from full medical ethical approval by the CMO region Arnhem – Nijmegen according to the Dutch Law. The study protocol was subsequently reviewed and approved by de Ethical Committee of the BMS faculty of the University of Twente. Therefore, all substantial amendments will only be notified to the Ethical Committee of the BMS faculty of the University of Twente. Non-substantial amendments will be recorded and filed by the sponsor. The online trial registry will be updated accordingly, and changes will be communicated in the publication of the results of this study.

#### Dissemination plans {31a}

It is our intention to publish the findings of the study in (medical) scientific journals and to present them at scientific meetings. The responsibility for publications and presentations lies with the investigators. Only those investigators making a significant contribution to the study design and/or the collection, analysis, or interpretation of the study data will be eligible for authorship. No restrictions regarding the public disclosure and publication of the research data have, or will be made, by the funder.

## Discussion

Currently, there is little knowledge about the effectiveness of interventions, and the cost-effectiveness of the novel care approach (motivational interviewing combined with digital shoe-fitting) has not been studied at all [29]. Therefore the aim of this randomized controlled trial is to assess the (cost-)effectiveness of this novel care approach compared to usual care in terms of adherence to wearing orthopedic shoes and ulcer prevention. Since the start of including the first participants in our study, we improved and modified our initial protocol based on operational and logistic issues and new insights; the most important changes are described and explained below.

As in any trial, patient recruitment is crucial. Based on the abovementioned power analysis the required sample size, including the loss to follow-up, for this study was estimated at 220 participants. These participants were to be included in a period of 9 months throughout The Netherlands. However, for practical reasons, it was not feasible to include throughout The Netherlands, and therefore inclusion will only take place in the east of The Netherlands. Because of this change and the outbreak of COVID-19, the goal of 220 participants is no longer realistic with the initially defined criteria and design in the intended period. In order to include as many participants as possible, we were forced to make some changes to the original study protocol.

First, we no longer include only patients receiving their first pair of orthopedic shoes, but we also include patients who already had orthopedic shoes and are eligible for a new pair of orthopedic shoes. Therefore, the pedorthist is now also actively involved in participant recruitment.

Second, due to this new role of the pedorthist, the background assignment of the treating podiatrist (being trained in MI or not) no longer determines the shoe-fitting procedure by the pedorthist. Each pedorthist has his/her own procedure of shoe-fitting: the new digital shoe-fitting procedure or the casting-based shoe-fitting procedure. In addition, the existing last is also used for an extra pair of orthopedic shoes, and thereby the shoe-fitting procedure is already determined for each subsequent pair of orthopedic shoes. However, the background assignment of the treating podiatrist remains leading over the shoe-fitting procedure with regard to which group the participant belongs to (intervention or control group), because most participants have been treated by the same podiatrist for years and we do not want to change that for this study. The participants in the intervention group will have an appointment with their podiatrist or one of the other MI-trained podiatrists to perform motivational interviewing before or as soon as possible after they receive their first or new pair of orthopedic shoes.

Last, the second group of 12 additional podiatrists received MI training, and 16 podiatrists have been added to the control group to further increase inclusion. Because the inclusion in the intervention group lagged behind the control group, the second group of podiatrists, who received MI training, consists of the podiatrists who see most patients with diabetes. This is in contrast to the original group of podiatrists, who were assigned to one of the groups based on stratified randomization.

In addition, a few small changes to the number of study consultations have also been made: 1) We reduced the number of six consultations that the participants would have with one of the investigators by two consultations. The investigator sees the participant at the delivery of the orthopedic shoes (T0), and 3 months (T1), 6 months (T2), 9 months (T3), and 12 months (T4) after the participant received their orthopedic shoes. 2) As a result of this change, the participants will receive the activity monitor during T1 (3 months) and T2 (6 months) instead of T1 (2 to 4 weeks) and T3 (4 months) as shown in Table 2. 3) All participants will be asked to fill in the MOS 6 months after receiving their orthopedic shoes, also the participants who will be approached for a second in-depth interview. 4) During every consultation with one of the investigators, the participant will be asked if they have an ulcer or have had an ulcer in the last 3 months. If so, the participant will be asked to fill in the iPCQ.

In conclusion, this trial aims to assess the (cost-)effectiveness of this novel care approach compared to usual care in terms of adherence to orthopedic shoes and ulcer prevention. The outcomes of this trial will generate insights into the socio-economic impact of the novel care approach on adherence to orthopedic shoes. These are crucial steps toward better ulcer prevention in high-risk diabetes patients.

## Trial status

The first version of this study protocol (22 January 2019) was registered at The Netherlands Trial Register (registration number NL7710, https://www.trialregister.nl/trial/7710) on 6 May 2019. The trial commenced recruitment in July 2019. Inclusion is currently ongoing and expected to be completed in December 2020.

## Abbreviations

AVG: Algemene verordening gegevensbescherming
BMI: Body mass index
BMS Lab: Behavioural management and social sciences lab
CEA: Cost-effectiveness analysis
CMO: Commissie mensgebonden onderzoek
COVID: Coronavirus disease
DANS: Data archiving and network services
EQ-5D-5L: 5-Level euroqol quality of life scale
EU: European union
GLMM: Generalized linear mixed model
ICER: Incremental cost-effectiveness ratio
iMCQ: iMTA medical consumption questionnaire
iMTA: Institute for medical technology assessment
iPCQ: iMTA productivity cost questionnaire
IWGDF: International working group on the diabetic foot
LISA: Library, ICT Services & archive
LOPS: Loss of protective sensation
MI: Motivational interviewing
MINT: Motivational interviewing network of trainers
MITI: Motivational interviewing treatment integrity
MOS: Monitor orthopedic shoes
PAD: Peripheral artery disease
QALY: Quality-adjusted life year
RAND-36: Research and development 36-item health survey
SPIRIT: Standard protocol items: recommendations for interventional trials
TIIM: The incredible intervention machine
UAVG: Uitvoeringswet AVG
USA: United States of America
WMO: Medical research involving human subjects act; in dutch: wet medisch-wetenschappelijk onderzoek met mensen
